# Deceptive Caffeine Shows Limited Impact on Short-Term Neuromuscular Performance

**DOI:** 10.3390/nu18020295

**Published:** 2026-01-17

**Authors:** Fernando Valero, Christian José Viudez, Sergio De la Calle, Fernando González-Mohíno, Juan José Salinero

**Affiliations:** Sport Training Laboratory, Faculty of Sport Sciences, University of Castilla-La Mancha, 45071 Toledo, Spain; fernando.valero1@alu.uclm.es (F.V.); fernando.gmayoralas@uclm.es (F.G.-M.)

**Keywords:** placebo, belief, exercise performance, jump, bench press

## Abstract

**Background**: Deceptive caffeine ingestion has shown inconsistent effects as an ergogenic aid for short-term exercises. **Objective:** Therefore, the aim of this study was to evaluate the potential placebo effect of deceptive caffeine ingestion on short-term performance during countermovement and repeated-jump tests, as well as bench press throw and bench press-to-failure assessments, and to document any associated side effects. **Methods:** A repeated, randomized, and counterbalanced design was implemented to compare the effects of ingesting a placebo claimed to be caffeine with a control condition in which no substance was consumed. Twenty-five physically active young adults (17 men and 8 women) completed a countermovement jump (CMJ) test, a 15 s repeated-jump test, bench press throws at 25%, 50%, and 75% of 1RM, and a bench press-to-failure test at 75% of 1RM and also completed a questionnaire regarding potential side effects. **Results:** Performance was similar between the placebo and control in the CMJ (38.1 ± 6.8 vs. 37.5 ± 6.8 cm; *p* = 0.225; *d* = 0.25) and in the 15 s repeated-jump test (*p* > 0.05; trivial–small effects). In the bench press throw, propulsive mean velocity did not differ at 25% 1RM (*p* = 0.296; *d* = 0.23) or 50% 1RM (*p* = 0.626; *d* = 0.10). However, deceptive caffeine ingestion increased propulsive mean velocity at 75% 1RM (0.500 ± 0.131 vs. 0.480 ± 0.131 m/s; *p* = 0.024; *d* = 0.48) and increased repetitions to failure at the same load (11.9 ± 3.7 vs. 11.0 ± 3.1; *p* = 0.047; *d* = 0.42). Mean velocity during the first 3 repetitions tended to be higher with the placebo (*p* = 0.064; *d* = 0.39), while final repetitions were similar (*p* = 0.469; *d* = 0.15). The most common side effects were increased activeness (34.8%) and nervousness (17.4%). **Conclusions:** In summary, deceptive caffeine ingestion had minimal impact on jump and ballistic bench press performance in physically active individuals. However, repetitions to failure were improved by ≈1 repetition (+8.2%). These findings suggest that the placebo effect of caffeine is unlikely to serve as a reliable strategy for enhancing short-term exercise performance.

## 1. Introduction

The placebo effect—an improvement driven solely by the belief in having ingested a beneficial substance—has been widely examined in medicine, psychology, and neuroscience, where it is recognized as a powerful neurobiological phenomenon [1,2,3,4,5,6]. In sports science, accumulating evidence indicates that placebos can elicit small to moderate performance enhancements, even in the absence of an active substance [7,8,9,10,11]. This potential to enhance exercise outcomes has stimulated growing interest in placebo responses related to nutritional and mechanical ergogenic aids [7]. A central factor of the placebo effect is the athlete’s expectation regarding the supposed ergogenic aid [2], as expectancy manipulations have been shown to substantially alter perceived exertion without necessarily inducing concomitant improvements in objective performance outcomes [11,12]. In this context, caffeine represents an optimal candidate for deceptive-placebo research, as it is widely used by elite athletes [13,14,15] and its ergogenic effects are well documented in several systematic reviews focusing on endurance activities and strength–power performance [16,17,18,19].

While the ergogenic effects of caffeine on endurance performance have been well documented for decades [16,20,21], its potential benefits for muscle strength were dismissed for several years [22]. Nowadays, there is strong evidence that acute caffeine intake (typically 3–6 mg/kg) enhances maximal strength, power output, and muscular endurance [17]. This classification of caffeine as an ergogenic aid is supported by numerous meta-analyses, including an umbrella review, showing consistent improvements in strength, rate of force development, velocity, and power across resistance exercises [17,19,23,24]. Specifically, in tests such as the bench press [25,26,27,28], repetitions to failure [27,29], countermovement jump (CMJ) [30,31,32], or repeated jumps [31], multiple studies have demonstrated that caffeine improves performance.

However, caffeine consumption is associated with some adverse side effects, such as insomnia, nervousness, or gastrointestinal discomfort [33,34,35,36]. For this reason, using caffeine as an ergogenic aid in sports scenarios could negatively impact performance if these side effects occur. In such cases, the use of placebos could minimize these side effects and potentially improve performance in a similar way. In fact, some studies have documented comparable improvements with both deceptive and actual ingestion of a moderate dose of caffeine during a 1000 m running test in trained athletes [37]. Nevertheless, in explosive exercises such as jumps, other studies have found that although ingesting a placebo or caffeine may enhance CMJ performance compared with a control condition, the effects of caffeine versus control appear greater than those of placebo versus control [30]. Moreover, some studies did not find significant effects on jump performance when deceptive caffeine was administered [38,39]. These contradictory results may be related to differences in study design, such as the expectancy of ingesting a higher caffeine dose (e.g., 6 mg/kg [30] vs. 3 mg/kg [38]), type of exercise (CMJ [30,38] vs. triple jump from standing [39]), or participant characteristics (recreational [30,38] vs. trained athletes [39]). Thus, the results are somewhat contradictory, highlighting the need for further research in this area. Similarly, in other power–strength tests, such as ballistic bench press at different loads, contradictory results have been found [40,41]. Both Costa et al. [40] and Ortiz et al. [41] found improvement in propulsive mean velocity at 50% of 1RM, but not at higher loads (60 to 90% 1RM). Notably, none of these studies included loads below 50% 1RM, leaving this range unexplored.

Additionally, some authors have suggested that the use of placebos could raise ethical concerns [42], and adverse effects may sometimes arise that resemble, or are associated with, the typical side effects of the actual substance (i.e., caffeine) [38,39]. Therefore, before using placebos to enhance sport performance, it is necessary to ensure their efficacy and consider potential concerns, such as ethical issues, economic cost, and possible side effects.

Accordingly, the aim of this study was to examine the potential placebo effect of deceptive caffeine ingestion on neuromuscular performance and any associated side effects. The specific objectives were to evaluate the placebo-induced changes in muscular power output (i.e., ballistic bench press at 25%, 50%, and 75% of 1RM, and CMJ) and repetitions to failure (i.e., bench press to failure at 75% of 1RM and a 15 s repeated-jump test).

## 2. Materials and Methods

### 2.1. Design

A randomized, counterbalanced, repeated-measures experimental design was used to examine the potential placebo effect of deceptive caffeine ingestion on neuromuscular performance. The experimental protocol was reviewed and approved by a local ethics committee (Ref. 28.1.2021CEI-UCJC).

### 2.2. Participants

Based on an a priori power analysis for a paired-samples *t*-test, assuming a moderate effect size (*d* = 0.6), a two-tailed α = 0.05, and a desired statistical power of 0.80, the required sample size was 24 participants (G*Power 3.1). Therefore, 25 participants were recruited to account for potential dropouts and ensure adequate statistical power. They were physically active young participants (17 men and 8 women; age: 22 ± 2 years; height: 170 ± 9 cm; weight: 68.0 ± 11.5 kg; 1RM: 67.4 ± 22.5 kg) who volunteered to complete the experimental procedures. Inclusion criteria were being young adults (18–35 years) and physically active, defined as engaging in at least three sessions of structured exercise per week (e.g., resistance or aerobic training) over the previous six months, and having no pathologies or physical limitations that could affect performance in the tests performed. All subjects participated voluntarily in this study after signing a written informed consent form and being informed of the experimental procedures.

### 2.3. Procedures

Before the experimental sessions, participants’ one-repetition maximum (1RM) for the bench press was estimated using the Brzycki formula [43], and body weight was measured to the nearest 0.1 kg with a calibrated digital scale (Seca Ltd., Hamburg, Germany). Participants were informed that body weight was necessary to calculate an adjusted caffeine dose (3 mg·kg^−1^ of body mass, based on studies indicating this dose is optimal for performance [25,26,31,32]). At that moment, information about the ergogenic effects of caffeine was provided to reinforce participants’ expectancy. Specifically, participants were informed about previous studies reporting performance improvements in jumping tasks and in bench press exercises performed at different loads. This information was conveyed by one of the authors (JJS), who has extensive research experience in caffeine and sports performance and was known to the participants, thereby further reinforcing their expectancy regarding the potential ergogenic effects of caffeine. After this, they were instructed to avoid intense exercise, caffeine, or energy drinks for 24 h before each session and to replicate their nutritional and rest habits on both days. All tests were conducted over two sessions, both held in the morning, scheduled at the same time of day to minimize potential circadian influences [44]. One session involved the ingestion of a placebo substance (100 mg of cornflour), administered in an opaque capsule 60 min before testing (placebo), while the other session involved no ingestion (control). The order of the experimental conditions was counterbalanced, with 12 participants completing the placebo session first and 13 participants completing the control session first. Both sessions were interspersed by 72 h to ensure proper recovery and to minimize hormonal fluctuations in women.

**Figure 1:**
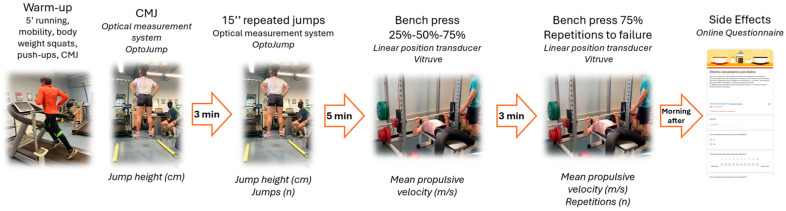
Schematic overview of the experimental procedure and measurement timeline, indicating the tests performed, the instruments used (upper part), and the variables analyzed (lower part).

Before each experimental session, participants completed a standardized warm-up of 5 min of self-selected moderate-intensity running on a treadmill (Excite 500, Technogym, Madrid, Spain), joint mobility exercises, 10 body weight squats, and 10 push-ups. A specific warm-up of 3 submaximal CMJs was performed before the CMJ test. The order of physical performance tests in each session was as follows (Figure 1):

Countermovement Jump. Three attempts were performed, with a 1 min rest between repetitions. Participants were instructed to jump as high as possible, and the highest jump was used for analysis. Arms were placed on the hips during the jump tests to avoid the influence of the arm swing on jump height, and a researcher verified that take-off and landing were performed in a correct position. Jump height was obtained using an optical measurement system (Optojump-next, Microgate, Bolzano, Italy). This device is a reliable and valid device to measure jump performance [45]. A 3 min rest was given before the next test.

15 s Repeated-Jump Test. Participants were required to perform all jumps at maximum height for 15 s. Participants were instructed to “jump as fast and as high as you can in 15 s”. Only one attempt was performed. Arms were placed on the hips during all jumps as in the CMJ. The jump height for each jump was recorded using the same optical measurement system. One participant reported knee pain during the repeated-jump test and stopped it. So, their data were excluded from the analysis in this test.

Bench Press Throws at 25%, 50%, and 75% of 1RM: This test was performed on a guided Smith machine (Multipower M-433, Salter SA, Barcelona, Spain). Two attempts were performed for each load, with a 1 min rest between repetitions and between loads. For each repetition, mean propulsive velocity was registered using a linear position transducer (Vitruve encoder, Vitruve Fit, Madrid, Spain). The reliability and validity of this type of device have been previously demonstrated [46]. A 3 min rest was given before the next test.

Bench Press to Failure at 75% of 1RM. Participants completed as many repetitions as possible at a self-selected pace until fatigue. Velocity data for each repetition and the number of repetitions were recorded using the same linear position transducer.

The morning following each session, participants were sent an online questionnaire to assess the potential side effects they may have experienced, such as irritability, increased urine production, difficulty sleeping, and gastrointestinal issues. This was performed only the morning after placebo ingestion. This questionnaire had previously been used to measure the side effects derived from deceptive caffeine consumption [38]. Participants who did not fill out the online form in time were removed from the analysis. This applied to 2 participants, so data from 23 participants were included in this variable.

Once data collection was completed, all participants were debriefed regarding the true aim of the study and the use of a placebo.

### 2.4. Data Analysis

Data are presented as mean ± SD. Normality was checked using the Shapiro–Wilk test, and differences between the placebo and control conditions were assessed with the paired *t*-test (with a non-parametric Wilcoxon test used in cases of non-normal distribution). Additionally, effect sizes (ES) were calculated by using Cohen’s d, and they were interpreted according to the following thresholds: <0.20 trivial, ≥0.20–0.59 small, ≥0.60–1.19 moderate, ≥1.20–1.99 large, and ≥2.00 very large [47]. The smallest worthwhile change (SWC) was calculated as 0.2 × the SD of the control condition for each variable [48]. Data on side effects are presented as mean ± SD and percentages to represent the proportion of athletes who reported each side effect. In all statistical analyses, the significance level was established at *p* < 0.05. All calculations were performed with JASP (v0.95.4 for Windows).

## 3. Results

### 3.1. Jump Performance

**Figure 2:**
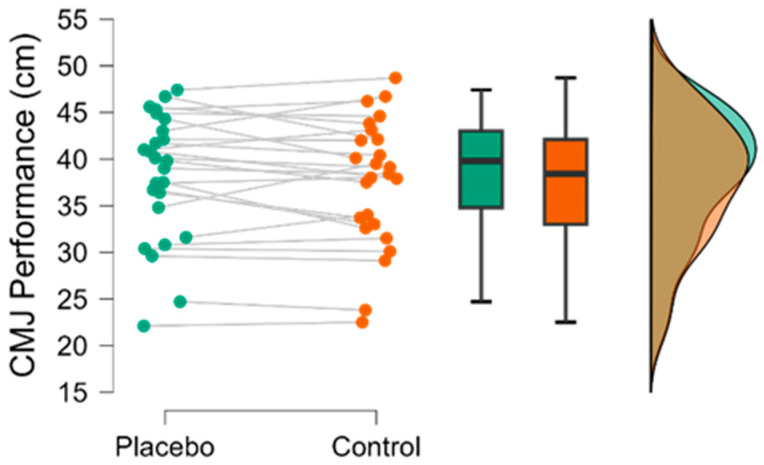
CMJ performance under both conditions. Individual data points with lines connecting paired observations, box-plot, and a density plot of the distribution of 25 participants in the placebo and control conditions.

Figure 2 illustrates the individual and group performance in the CMJ test. No statistically significant differences were observed between the deceptive caffeine ingestion (38.1 ± 6.8 cm) and control condition (37.5 ± 6.8 cm; *p* = 0.225; small effect *d* = 0.25). Among the 25 participants, 8 exhibited improvements exceeding the SWC in jump height following deceptive caffeine ingestion (+3.4 ± 1.1 cm), 4 showed decrements greater than the SWC (−3.2 ± 1.2 cm), and the remaining participants displayed no substantial change.

In the 15 s repeated-jump test (Table 1), participants demonstrated similar performance across both experimental conditions. The total number of jumps was nearly identical between the placebo (13 ± 3 jumps) and control (13 ± 3 jumps) conditions (*p* = 0.187; small effect *d* = 0.26). Analysis of the first (1st to 3rd) and last three jumps revealed no meaningful differences in jump height. Jump height in the first jumps was comparable between the placebo and control conditions (31.2 ± 6.1 vs. 30.6 ± 5.9 cm; *p* = 0.280; small effect *d* = 0.23). Similarly, jump height in the final jumps was almost identical between conditions (27.9 ± 5.6 cm placebo vs. 27.8 ± 5.7 cm control; *p* = 0.867; trivial effect *d* = 0.04).

### 3.2. Bench Press Throws at 25–50–75% of 1RM

**Figure 3:**
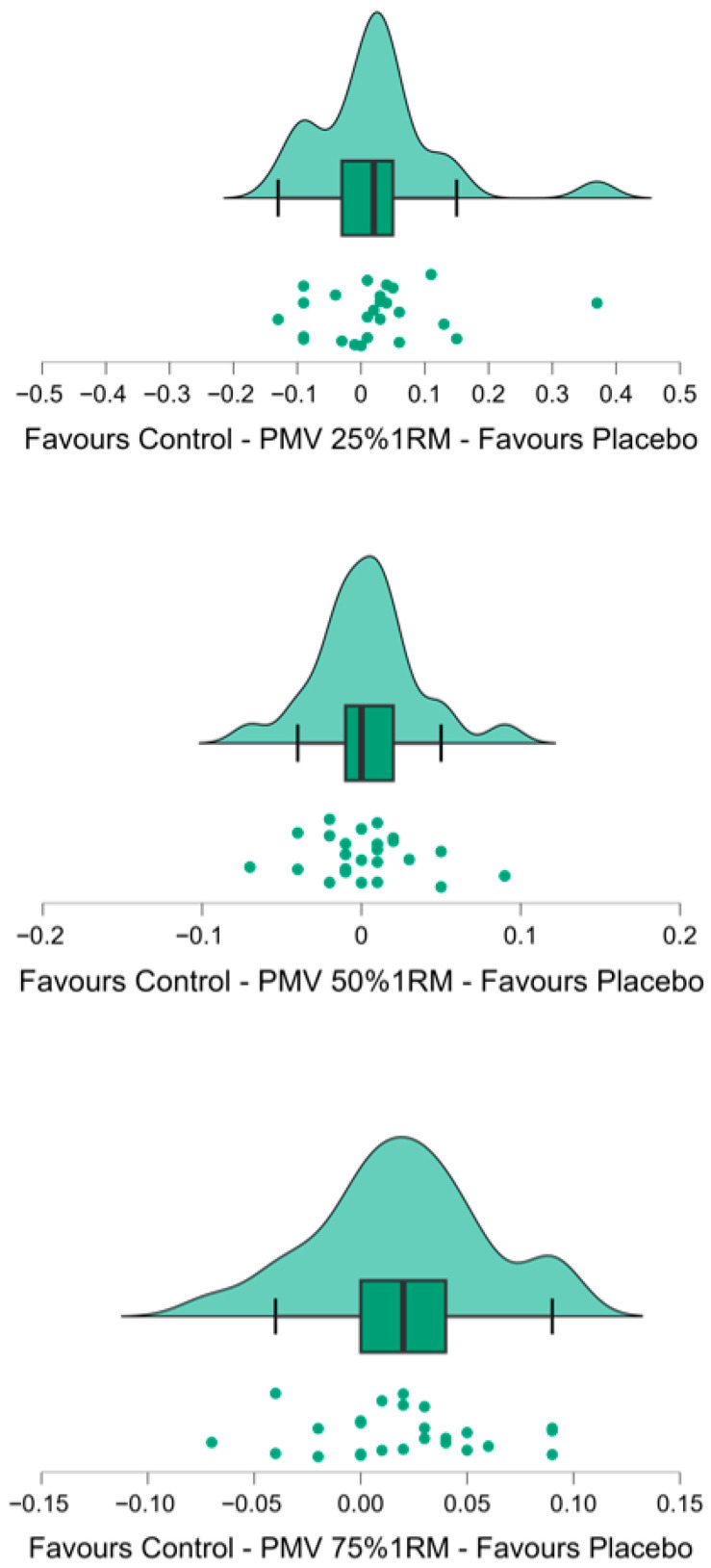
Raincloud plot illustrating the distribution of individual differences between the placebo and control conditions in Mean VPM at 25, 50 and 75% 1RM.The half-violin shape represents the smoothed distribution density of the difference scores. The boxplot displays the interquartile range (IQR), with the median indicated by the central line and the whiskers representing 1.5 × IQR. Each dot corresponds to an individual participant’s difference value. Negative values indicate lower scores in the placebo condition compared to control, whereas positive values indicate higher placebo scores.

In the bench press throw (Table 2, Figure 3), propulsive mean velocity was similar between conditions at both 25% 1RM (*p* = 0.296; small effect *d* = 0.23) and 50% 1RM (*p* = 0.626; trivial effect *d* = 0.10). However, deceptive caffeine ingestion significantly increased propulsive mean velocity at 75% 1RM (0.500 ± 0.131 m/s placebo vs. 0.480 ± 0.131 m/s control; *p* = 0.024; small effect *d* = 0.48). Eleven participants showed improvements exceeding the SWC (+0.055 ± 0.025 m/s), 3 participants experienced declines greater than the SWC (−0.050 ± 0.017 m/s), and the remaining 11 showed no meaningful change. In addition, deceptive caffeine ingestion increased repetitions to failure at 75% 1RM (11.9 ± 3.7 reps. with placebo vs. 11.0 ± 3.1 reps. with control; *p* = 0.047; small effect *d* = 0.42). Regarding this test, 15 individuals improved (2.1 ± 2.0 reps.), and 7 declined (−1.3 ± 0.8 reps.), both exceeding the SWC thresholds. Mean velocity in the first 3 repetitions tended to be greater with deceptive caffeine ingestion (0.413 ± 0.103 m/s vs. 0.398 ± 0.103 m/s; *p* = 0.064; small effect *d* = 0.39) while it was similar in the last 3 repetitions (0.201 ± 0.046 m/s vs. 0.206 ± 0.047 m/s; *p* = 0.469; trivial effect *d* = 0.15).

### 3.3. Side Effects

The most common side effects reported by participants were increased activeness (8 out of 23, 34.8% of participants who fulfilled the online form) and nervousness (4 out of 23, 17.4%), with minimal reports of other adverse side effects (Table 3).

## 4. Discussion

The present study aimed to examine the effects of deceptive caffeine ingestion on jump performance and bench press performance across different loads. For this objective, 25 participants completed 2 experimental conditions (deceptive caffeine and a control condition without any substance ingested). Overall, the findings revealed limited placebo effects on neuromuscular performance. No significant differences were observed in CMJ performance between conditions, and performance in the 15 s repeated-jump test was also very similar across conditions, indicating that the expectancy effect did not meaningfully influence jump performance. In contrast, deceptive caffeine ingestion produced small but statistically significant improvements in propulsive mean velocity and repetitions to failure during the bench press exercise at 75% 1RM, while no meaningful changes were observed at lower loads.

Regarding jump performance, the deceptive caffeine condition resulted in improvements greater than SWC on 8 of 25 participants in CMJ height, and these individual responses did not translate into significant group effects (Figure 2). This reinforces the idea that placebo-induced alterations in explosive lower-body performance may be modest and highly individual. In the 15 s repeated-jump test, neither the total number of jumps nor jump height in the initial or final repetitions differed meaningfully between conditions (Table 1). These findings suggest that perceived caffeine ingestion does not substantially influence repeated stretch–shortening cycle actions or fatigue-related decrements in jump height over short-duration efforts. Previous research about deceptive caffeine ingestion on jump performance has found contradictory results. Grgic, Venier y Mikulic [30] found that administering a placebo (as compared to a control condition) was ergogenic for increasing vertical jump height. Nevertheless, Del Coso et al. [38] found no differences between deceptive caffeine ingestion and control condition. Predictably, methodological aspects such as the information provided to participants, their beliefs about the substance, contextual variables, and participant characteristics could have influenced the outcomes of these studies.

Previous studies reported that deceptive caffeine ingestion enhanced performance at ballistic bench press only at 50% 1RM [40,41]; they found no improvements at higher loads, and none of these studies examined lighter loads than 50% 1RM. To address this gap, our study tested three intensities—the commonly used 50% 1RM, one lower load (25% 1RM), and one higher load (75% 1RM)—to examine whether expectancy effects are load-dependent. Contrary to earlier reports, we observed no improvement at 50% 1RM, while an ergogenic placebo effect emerged at the higher load. These findings challenge previous assumptions and highlight the need to clarify the load-dependent nature of placebo responses in resistance exercise. Complementing these results, we also observed an increased number of repetitions to failure at 75% 1RM, consistent with previous reports showing enhanced repetitions under deceptive caffeine conditions [11,49]. Interestingly, mean propulsive velocity during the initial repetitions showed a trend toward significance (*p* = 0.06, effect size = 0.39), indicating that participants began the set moving the bar faster. Despite this, bar velocity during the final repetitions was similar across conditions, even though participants completed, on average, ≈1 additional repetition. Together, these findings suggest that expectancy may exert stronger influences when tasks require sustained effort or approach volitional failure, rather than during brief, high-intensity actions. Mechanistically, the effects may reflect changes in motivation, perceived energy, and a transient increase in perceived readiness, as suggested by the initial rise in movement velocity. Aligned with these performance outcomes, subjective responses also reflected expectancy-driven effects. Activeness or perceived stimulation emerged as the most commonly reported response, with 34.8% of participants indicating that they felt more energetic when they believed they had ingested caffeine. This aligns with previous placebo-based strength research showing that expectancy alone may modify perceived activation levels [41]. Additionally, 17.4% of participants reported increased nervousness under the supposed caffeine condition. Although these symptoms were mild and short-lived, nervousness constitutes a potentially negative effect—particularly in athletic contexts where excessive arousal could impair technical execution or decision-making. Taken together, these findings suggest that placebo ingestion does not induce meaningful adverse side effects within the context of acute resistance-training tests. However, some expectancy-driven responses—such as increased nervousness—may have practical implications. Therefore, while caffeine-related placebos appear safe, their use in sports settings should be approached with caution, especially when optimal performance depends on carefully regulating arousal levels.

This study has several limitations. First, the sample consisted of physically active individuals, which may limit the generalizability of the findings to trained athletes or sedentary populations. Second, the placebo manipulation was based solely on deceptive information rather than on comparisons with different actual caffeine doses, which prevents distinguishing the relative contributions of pharmacological and expectancy effects. Third, the performance tests targeted specific resistance exercises, and the results may not translate to sport-specific or more technical movements, where motor skill demands could alter the placebo response. Lastly, individual differences in caffeine-related beliefs, habitual caffeine intake, and susceptibility to placebo effects were not controlled. Participants’ expectancy regarding the effects of the substance was not specifically assessed. Although participants were informed about the potential ergogenic effects of caffeine ingestion on performance in tests similar to those performed in this study, their individual beliefs or expectations about these effects were not formally measured, potentially contributing to the inter-individual variability observed among participants.

## 5. Conclusions

In summary, deceptive caffeine ingestion had a small impact on jump and ballistic bench press performance in physically active individuals. These findings suggest that the placebo effect of caffeine is unlikely to serve as a reliable strategy for enhancing short-term exercise performance.

## Figures and Tables

**Table 1:** 15 s repeated-jump test.

	Control	Placebo	*p*	Effect Size *d*
Jump number	13 ± 3	13 ± 3	0.187	0.26
Mean height for the first 3 jumps (cm)	30.6 ± 6.1	31.2 ± 6.1	0.280	0.28
Mean height for the last 3 jumps (cm)	27.8 ± 5.7	27.9 ± 5.6	0.867	0.04

**Table 2:** Bench press throws at 25%, 50%, and 75% 1RM.

	Control	Placebo	*p*	Effect Size *d*
Bench press throw				
Propulsive mean velocity 25% (m·s^−1^)	1.245 ± 0.201	1.268 ± 0.237	0.296	0.23
Propulsive mean velocity 50% (m·s^−1^)	0.785 ± 0.145	0.788 ± 0.142	0.626	0.10
Propulsive mean velocity 75% (m·s^−1^)	0.480 ± 0.131	0.500 ± 0.131	0.024	0.48
Bench press: repetitions to failure				
Repetitions (*n*)	11.0 ± 3.1	11.9 ± 3.7	0.045	0.42
Mean first 3 repetitions (m·s^−1^)	0.398 ± 0.103	0.413 ± 0.103	0.064	0.39
Mean last 3 repetitions (m·s^−1^)	0.206 ± 0.047	0.201 ± 0.046	0.469	0.15

**Table 3:** Perceived side effects after deceptive caffeine ingestion.

	Mean ± SD	Affirmative Responses (%)
Nervousness	2.1 ± 1.8	17.4
Activeness	3.2 ± 2.3	34.8
Irritable	1.6 ± 1.3	8.7
Muscular pain	1.5 ± 1.0	4.3
Headache	1.4 ± 1.3	4.3
Gastrointestinal discomfort	1.9 ± 1.8	8.7
Diuresis	1.5 ± 1.2	4.3
Sleep disorders	1.65 ± 1.3	4.3

## Data Availability

The original contributions presented in this study are included in the article. Further inquiries can be directed to the corresponding author.
